# Evaluation of Soluble Urokinase Plasminogen Activator Receptor in COVID-19 Patients

**DOI:** 10.3390/jcm13216340

**Published:** 2024-10-23

**Authors:** Simona Arientová, Kateřina Matúšková, Oldřich Bartoš, Ondřej Beran, Michal Holub

**Affiliations:** 1 Department of Infectious Diseases, Military University Hospital Prague and First Faculty of Medicine of Charles University, 16902 Prague, Czech Republic; simona.arientova@uvn.cz (S.A.); matuskova.katerina@uvn.cz (K.M.); ondrej.beran@uvn.cz (O.B.); 2 Military Health Institute, Military Medical Agency, 16200 Prague, Czech Republic; 3 Ústřední Vojenská Nemocnice—Vojenská Fakultní Nemonice Praha, U Vojenské Nemocnice 1200, Praha 6-Břevnov, 16902 Prague, Czech Republic

**Keywords:** COVID-19, urokinase-type plasminogen activator receptor, C-reactive protein, interleukin-6, procalcitonin

## Abstract

**Background/Objectives:** This retrospective study analyzed soluble urokinase plasminogen activator receptor (suPAR) plasma levels alongside routine inflammatory markers, including the neutrophil-to-lymphocyte count ratio, C-reactive protein (CRP), interleukin-6 (IL-6), procalcitonin (PCT), and D-dimers in COVID-19 patients hospitalized during the Omicron wave of the pandemic. **Methods:** We measured plasma suPAR levels using a suPARnostic^®^ Quick Triage kit. We divided COVID-19 patients into two groups based on the severity of SARS-CoV-2 infection according to the National Institutes of Health (NIH) criteria. The logistic regression analysis tested the predictive value of the biomarkers. **Results:** We evaluated 160 consecutive COVID-19 patients hospitalized between January and August 2022. The cohort exhibited a high incidence of comorbidities, with an in-hospital mortality rate of 5.6%. Upon admission, the median suPAR plasma levels were not significantly different between patients with mild COVID-19 (n = 110) and those with moderate/severe disease (n = 50), with 7.25 ng/mL and 7.55 ng/mL, respectively. We observed significant differences (*p* < 0.01) between the groups for CRP and IL-6 levels that were higher in moderate/severe disease than in mild infection. Additionally, suPAR plasma levels were above the normal range (0–2.00 ng/mL) in all patients, with a significant positive correlation identified between suPAR levels and serum IL-6, PCT, and creatinine levels. **Conclusions:** These findings indicate that COVID-19 during the Omicron wave is strongly associated with elevated suPAR levels; however, these levels do not directly correlate with the severity of SARS-CoV-2 infection.

## 1. Introduction

Since the beginning of the coronavirus disease 2019 (COVID-19) pandemic, clinicians have used various biomarkers to assess the severity of SARS-CoV-2 infection. Thus, the number of circulating lymphocytes, their ratio to the number of circulating neutrophils (neutrophil-to-lymphocyte count ratio, NLCR), and the serum levels of ferritin, C-reactive protein (CRP), and interleukin (IL)-6 were included in the majority of diagnostic panels used in COVID-19 patients [1]. In addition to these biomarkers, serum levels of procalcitonin (PCT) together with D-dimer (DD) plasma levels are frequently measured in COVID-19 patients because PCT serum levels ≥ 0.5 μg/L are indicative of bacterial superinfection, and elevation of DD plasma levels may suggest thromboembolic disease [2,3].

Recently, soluble urokinase plasminogen activator receptor (suPAR), which plays a role in various inflammation-related diseases and infections, including acute pancreatitis, systemic inflammatory syndrome, renal failure, bacteremia, and sepsis, has gained significant attention due to its potential value in predicting severe respiratory failure (SRF) associated with COVID-19 [4,5,6]. In line with this relatively small study involving 21 Greek patients with SRF, an observational study conducted in Denmark between March and April 2020, which included 386 patients with SARS-CoV-2 infection, demonstrated the usefulness of suPAR serum concentrations for risk stratification of severe COVID-19. Patients with concentrations below 4 ng/mL had a higher likelihood of short hospital stays lasting 24 h than patients with suPAR concentrations above 6 ng/mL, with a significantly higher probability of hospitalization lasting up to 14 days [7]. Additionally, serum concentrations of suPAR analyzed during the second wave of COVID-19 in Italy in patients with severe SARS-CoV-2 infection showed significantly higher levels in those who died compared to survivors [8].

Subsequently, the multicenter randomized study called SAVE-MORE, which investigated the treatment of COVID-19 with anakinra, an IL-1α/β inhibitor, drew considerable attention. This study found that patients with suPAR plasma levels above 6 ng/mL treated with anakinra had better outcomes than those in the placebo group [9]. Based on these data, which suggested potential expansions of our therapeutic options, we included measurements of suPAR plasma levels in our routine diagnostic panel used with patients hospitalized for COVID-19. After obtaining significant data, we evaluated suPAR prognostic potential and routine biomarkers of patients admitted to our department with SARS-CoV-2 infection.

## 2. Materials and Methods

This retrospective single-center study was conducted in the Department of Infectious Diseases at the Military University Hospital in Prague, evaluating data from adult patients aged 18 and older hospitalized between 1 January and 30 April 2022. The study included patients with PCR-confirmed SARS-CoV-2 infection who required in-hospital care. We classified the severity of COVID-19 according to NIH criteria as mild, moderate, or severe. Mild cases involved various symptoms such as fever, cough, and loss of taste or smell without respiratory distress or abnormal chest imaging. In contrast, moderate cases showed lower respiratory disease with SpO_2_ ≥ 94%, and severe cases had SpO_2_ < 94%, PaO_2_/FiO_2_ < 300 mm Hg, a respiratory rate > 30 breaths/min, or lung infiltrates involving more than 50% of lung tissue based on abnormal chest imaging on high-resolution CT. For study purposes, we calculated the Charlson Comorbidity Index (CCI) and analyzed vaccination status and anti-SARS-CoV-2 antibodies and routine laboratory parameters, including suPAR, CRP, PCT, NLCR, IL-6, DD, and creatinine. The patient’s therapy followed NIH guidelines. We recorded complications and in-hospital mortality.

Patient peripheral blood samples for suPAR and NLCR were collected in VACCUETTE^®^ blood collection tubes with K3E EDTA (Greiner BioOne, Kremsmünster, Austria) and in VACUETTE^®^ blood collection Serum Clot Activator tubes (Greiner BioOne, Kremsmünster, Austria) for determinations of CRP, IL-6, PCT, and creatinine serum concentrations. We used VACUETTE^®^ coagulation tubes with sodium citrate for coagulation and DD tests (Greiner BioOne, Kremsmünster, Austria). Whole blood for suPAR analysis was centrifuged at 3000× *g* for 15 min, and plasma was transferred to marked tubes and stored at −20 °C. To quantify suPAR concentrations, we used suPARnostic^®^ Quick Triage for aLF Reader (ViroGates, Birkerød, Denmark) following the manufacturer’s protocol. The suPARnostic^®^ Quick Triage has a measuring range of 2–15 ng/mL, a detection limit of 1.0 ng/mL, and a quantification limit of 2.0 ng/mL. White and differential blood cell counts were routinely measured immediately after blood collection using the Sysmex N-10 system (Sysmex, Kobe, Japan). D-dimer levels were measured using the coagulation analyzer ACL TOP 550 CTS (ACL TOP Family, Bedford, MA, USA). C-reactive protein, PCT, and IL-6 were routinely analyzed immediately after sample collection using a commercially available immunoturbidimetric assay (CRP; Roche Diagnostics GmbH, Mannheim, Germany) and electrochemiluminescence immunoassay (PCT, IL-6 and creatinine; Roche Diagnostics GmbH, Mannheim, Germany) with a Cobas Pro integrated solution modular analyzer (Roche Diagnostics GmbH, Mannheim, Germany). SARS-CoV-2 infection was confirmed using polymerase chain reaction (PCR) testing of specimens obtained by nasopharyngeal swab (Alinity m Instrument; Alinity m SARS-CoV-2 Assay; Abbott Laboratories, Chicago, IL, USA). The discriminative test for SARS-CoV-2 variants was not routinely performed based on the extremely high prevalence (>95%) of the SARS-CoV-2 Omicron variant in the Czech Republic during the study period and decision No. 1/2022 of the biggest healthcare insurance company not to reimburse discriminative PCR (General Health Insurance Company of the Czech Republic), which was effective from 31 January 2022. We used a chemiluminescent assay CLIA (LIAISON XL, kit LIAISON SARS-CoV-2 TrimericS IgG; DiaSorin, Saluggia, Italy) for the measurement of antibodies against SARS-CoV-2.

A certified statistician performed all statistical procedures in R software version 4.1.3 (R Core Team, 2022). Continuous variables are expressed as the median and interquartile range [IQR]. The Shapiro–Wilk normality test tested the normality of the data. The correlation between individual continuous variables was estimated using Spearman’s correlation coefficient. We utilized multiple logistic regression to assess whether there was a relationship between the selected parameters (i.e., CCI, suPAR, CRP, PCT, NLCR, IL-6, and DD, and disease severity). Due to the feasibility and the limited number of observations/patients, we divided the patients into two categorical groups according to their disease severity: mild and moderate/severe disease at admission. The model was determined by a stepwise procedure using the step function and Wald test for subsequent evaluation. Nagelkerke statistics and pseudo-r-squared measures for various models also tested individual models. Moreover, we were looking for overdispersion, which would have indicated a poor fit between the model and the data. Furthermore, we examined whether there were any interactions between the individual variables.

## 3. Results

We enrolled 160 patients admitted to the hospital with SARS-CoV-2 infection. Table 1 shows the patients’ baseline characteristics, including biomarkers at admission. 

In all patients, PCR testing confirmed SARS-CoV-2 infection. Altogether, SARS-CoV-2 pneumonia was the reason for admission in 50 patients, and SARS-CoV-2 positivity and comorbidities led to the admission of 110 patients. Of the 105 patients treated with remdesivir, 36 had moderate/severe COVID-19, and 69 received remdesivir as prophylaxis for the severe course of SARS-CoV-2 infection. Of the patients with a severe course of SARS-CoV-2 infection, 31 patients were on conventional oxygen therapy, one patient was on high-flow nasal oxygen therapy, 13 patients with severe illness received dexamethasone, and 1 patient received biological therapy with sarilumab. A total of nine patients died during their hospital stay: three due to COVID-19 and six due to comorbidities.

**Figure 1:**
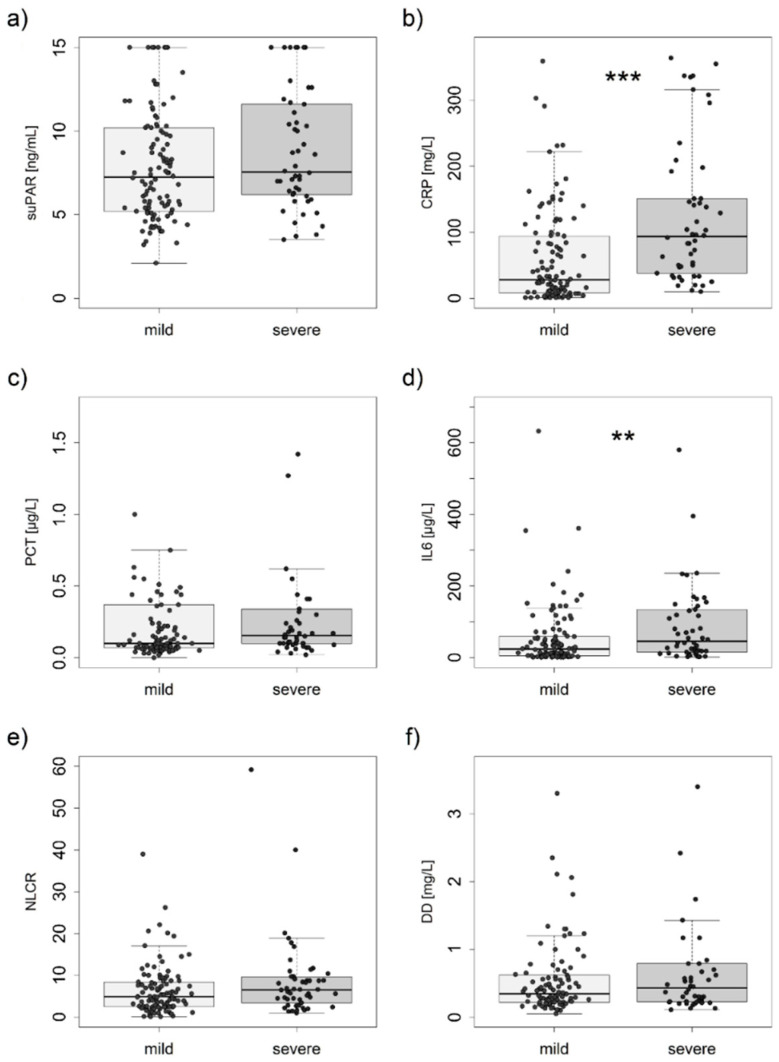
Comparison of biomarkers between mild and moderate/severe COVID-19. *** *p* value < 0.001; ** *p* value < 0.01; (**a**–**f**) suPAR—soluble urokinase plasminogen activator receptor; CRP—C-reactive protein; PCT—procalcitonin; IL—interleukin; NLCR—neutrophil-to-lymphocyte count ratio DD—D dimer; mild—mild course of COVID-19; severe—moderate/severe course of COVID-19; significant differences were observed only for CRP and IL-6.

The following parameters obtained at admission were above the norm: suPAR in 160 (100%), CRP in 140 (87.5%), PCT in 27 (16.88%), NLCR in 100 (62.50%), IL-6 in 121 (75.63%), DD in 113 (70.63%), and creatinine in 45 (28.1%) enrolled patients. In addition, 107 (66.88%) patients had suPAR > 6 ng/L, and 120 (75%) patients had at least one of the listed predisposing factors increasing suPAR plasma levels, such as chronic lung disease, chronic ischemic disease, hepatic cirrhosis, chronic kidney disease, or elevated creatinine [2,10,11]. When comparing patients in both groups, there were three deaths during the hospital stay in the group with a moderate/severe course of COVID-19 and six deaths in the group with mild disease (in-hospital mortality was 6% vs. 5.45%). Additionally, both groups had similar CCI—5.5 vs. 5.0 (*p* = 0.379), and there was no significant correlation with the severity of COVID-19 (*p* = 0.465). Regarding the biomarkers, in comparison with the patients with a mild course of COVID-19, the patients with a moderate/severe course had significantly elevated serum levels of CRP (*p* < 0.001) and IL-6 (*p* = 0.007). The groups significantly did not differ in suPAR, PCT, NLCR, and DD levels (Figure 1).

Table 2 shows the median values of individual biomarkers in patients with mild and moderate/severe COVID-19 at admission, together with the results of multiple logistic regression analysis demonstrating that CRP was only associated with the severity of COVID-19.

In addition, suPAR plasma levels demonstrated positive correlations with serum levels of IL-6 (r = 0.430; *p* < 0.001), PCT (r = 0.233; *p* = 0.003), and creatinine (r = 0.29; *p* < 0.001).

## 4. Discussion

This study assessed suPAR plasma levels alongside routine biomarkers in adult COVID-19 patients hospitalized during the pandemic wave driven by the SARS-CoV-2 Omicron variant. Our findings revealed a significant elevation of suPAR plasma levels in SARS-CoV-2 infection; they do not correlate with the severity of COVID-19, thus questioning the utility of suPAR as a prognostic marker in the context of the current course of COVID-19.

It is well known that suPAR is a biomarker for bacterial sepsis and renal failure [12]. Studies also support the measurement of suPAR blood levels for evaluating the severity of pneumococcal pneumonia and acute respiratory distress syndrome [13,14]. Furthermore, elevated plasma suPAR levels were the highest in critical cases of SARS-CoV-2 infection and in patients who did not survive the disease [15]. Similarly, Huang et al. [16] reported high plasma levels of an active form of suPAR in patients with severe and critical courses of COVID-19; however, nine asymptomatic SARS-CoV-2 carriers enrolled in their study demonstrated the highest plasma suPAR concentrations. In addition to the small number of these asymptomatic subjects, the authors suggested that the most likely reason for this unexpected finding was the analytical method used, which was an immunoassay rather than the generally recommended suPARnostic^®^ assays—the only approved and certified assays for in vitro diagnostics of suPAR levels in biological specimens [17].

However, our results are partially in line with the findings of elevated suPAR plasma levels in asymptomatic infections because we observed significantly increased plasma levels of suPAR in patients with a mild course of SARS-CoV-2 infection that were only slightly lower than those in patients with moderate/severe COVID-19. Since our study started just after the advent of the SARS-CoV-2 Omicron variant and the immunity of the population has changed throughout the pandemic, this probably had a significant impact on the characteristics of the affected population, the course of COVID-19, and the clinical utility of suPAR and other biomarkers. It became apparent early on during the pandemic wave caused by the SARS-CoV-2 Omicron variant that the population at risk of severe COVID-19 changed. When compared with the patients enrolled in the placebo group of the anakinra trial, our patients were older (mean age 78.6 vs. 61.9 years) and more polymorbid (mean CCI 5.9 vs. 2.2), but their in-hospital mortality was lower (5.6 vs. 6.9%) [9].

Regarding suPAR plasma levels, advanced age is associated with a mild increase. At the same time, common chronic diseases such as asthma and cardiovascular disease, along with acute conditions like acute exacerbation of chronic obstructive pulmonary disease and acute renal failure, lead to significantly elevated suPAR levels [10,11,17]. Consequently, the elevated baseline suPAR concentrations observed in our patients with a mild course of COVID-19 may reflect prevalent comorbidities and acute illnesses rather than the severity of SARS-CoV-2 infection. The differences between our cohort and the anakinra trial, where patients exhibited lower incidences of chronic respiratory diseases (4% vs. 25.6%), chronic cardiac diseases (13.4% vs. 48.8%), and baseline creatinine elevations (4.5% vs. 29.38%) support the role of comorbidities in suPAR elevations [9]. Additionally, the effect of renal dysfunction or failure on elevated suPAR levels is evident in their positive correlation with serum creatinine levels found in our study. The association of suPAR with elevated IL-6 serum levels in our cohort can reflect systemic inflammation caused by SARS-CoV-2 infection without a direct relationship between the two biomarkers, as demonstrated in an ex vivo model using whole blood stimulated with high concentrations of suPAR [18].

Of the other routinely used biomarkers, only CRP proved to be a predictor of the severity of COVID-19 in our cohort. Quin et al. [19] reported that serum CRP concentration was significantly higher in patients with severe COVID-19 than in those with a mild course of the disease, with median concentrations of 57.9 mg/L vs. 33.2 mg/L. Similarly, Villard et al. [20] found a significantly higher median serum CRP concentration of 152 mg/L in patients with a severe course of COVID-19 compared to 83 mg/L in those with a milder course. These findings align with our observations of lower CRP serum concentrations in the mild course of COVID-19 and higher levels in moderate to severe cases. Luan et al. [21] emphasized that CRP may play a pivotal role in the inflammation elicited by SARS-CoV-2 infection. Thus, CRP is considered an independent predictor of severe COVID-19, consistent with our finding of a significant association between CRP and disease severity. Although the two groups differed significantly in IL-6 levels, similar to CRP, IL-6 showed no predictive value for disease severity. Because many observations reached baseline values, particularly in group 1, the data distribution may be responsible for the lack of predictive value. At the same time, the overall variance remained comparable to group 2 (see Figure 1). This variance explains why a simple comparison distinguishes the two groups, but the predictive value of this marker is low.

In addition, patients with severe disease may have higher PCT levels than those with mild COVID-19, primarily due to bacterial superinfection [22]. It is well known that PCT levels in patients with viral infections are usually below the recommended cutoff for bacterial infection (i.e., <0.5 ng/mL). Therefore, PCT is a good predictor of bacterial superinfection, supporting the use of empirical antibiotic therapy in patients with COVID-19 [23,24]. In our study, an elevated PCT value > 0.5 ng/mL detected at admission triggered empirical antibiotic therapy in nearly 17% of the patients. It should also be emphasized that blood PCT levels may also hold predictive value, as COVID-19 patients with a mild disease course exhibited low PCT concentrations compared to those with a severe course of the infection (0.05 ± 0.05 vs. 0.44 ± 0.55 ng/mL) [25,26]. However, our study did not find any correlation between the severity of COVID-19 and PCT levels. This may reflect the fact that only a minority of the enrolled patients experienced severe disease that necessitated intensive oxygen therapy.

This study has several limitations. First, we started to measure suPAR plasma levels during the Omicron wave of COVID-19, a period characterized by high levels of population immunity and a different clinical profile compared to earlier phases of the pandemic dominated by more virulent strains like the original Wuhan strain and the Delta variant. These earlier strains were associated with higher mortality rates and more severe clinical presentations [27]. Second, the reasons for patient admissions differed from those in the earlier stages of the pandemic, significantly impacting the validity of the scoring system, which reflects the severity of SARS-CoV-2 infection rather than the overall severity of the patient’s conditions. Third, acute or chronic conditions associated with elevated suPAR plasma levels were the reason for the admission of 75% of our patients, which influenced our results. Nonetheless, this reflects the current SARS-CoV-2 infection affecting a polymorbid population.

## 5. Conclusions

The study offers a nuanced perspective on the role of suPAR in COVID-19. While we confirmed prior findings of elevated suPAR plasma levels in COVID-19 patients, our results did not reveal a correlation between suPAR levels and the severity of SARS-CoV-2 infection, particularly concerning the Omicron variant and populations with high comorbidity rates. This finding contrasts with earlier studies that demonstrated a solid predictive capacity of suPAR for severe disease outcomes. These discrepancies highlight the necessity of considering patient demographics, viral variants, and the presence of comorbidities when evaluating the significance of suPAR in the context of COVID-19.

## Figures and Tables

**Table 1:** Baseline characteristics of patients, including COVID-19 biomarkers at admission.

	n = 160	%
**Demographics**		
Age (years) *	78.6 (79; 34–100)	n.a.
Male	66	41
Charlson Comorbidity Index *	5.9 (5; 0–13)	n.a.
Full vaccination against COVID-19	117	73
In-hospital mortality	9	5.6
**Comorbidities**		
Asthma bronchiale	16	10
Chronic obstructive pulmonary disease	28	17.5
Chronic heart failure	78	48.8
Chronic renal failure	37	23.1
Hepatic cirrhosis	4	2.5
**NIH criteria for COVID-19 at admission**		
Mild	110	69
Moderate/severe	50	31
**Co-administered treatment**		
Remdesivir	105	65.6
Corticosteroids	24	15
Sarilumab	1	0.6
Antimicrobial treatment	75	46.9
Oxygen therapy	50	31.3
**Laboratory values at admission**		
**(normal values)**		
suPAR (<2 ng/mL) **	7.35 (5.400–10.300)	n.a.
CRP (<5 mg/L) **	46.5 (14.55–117.50)	n.a.
PCT (<0.5 µg/L) **	0.12 (0.0700–0.3625)	n.a.
NLCR (1.4–4) **	5.4 (2.600–8.700)	n.a.
IL-6 (<7 µg/L) **	31.1 (8.475–80.000)	n.a.
DD (<0.23 mg/L) **	0.37 (0.2200–0.6725)	n.a.
Creatinine (64–104 µmol/L) **	85.25 (71.30–118.00)	n.a.
SARS-CoV-2 IgG (<33.8 BAU/mL) **	2080 (457.75–2080.00) ***	n.a.

* Data are expressed as the means (medians; range); ** Data are expressed as the medians (interquartile range—IQR1–IQR3); n.a.—not applicable; COVID-19—coronavirus disease 2019; NIH—National Institute of Health; IQR—interquartile range; suPAR—soluble urokinase plasminogen activator receptor; CRP—C-reactive protein; PCT—procalcitonin; NLCR—neutrophil-to-lymphocyte count ratio; IL—interleukin; DD—D dimer; SARS-CoV-2 IgG—IgG antibodies to SARS-CoV-2; *** Used data from 158 patients.

**Table 2:** Association between analyzed biomarkers and COVID-19 severity at admission.

Biomarkers	Mild COVID-19	Moderate/Severe COVID-19	*p* Value **
(Normal Values)	(n = 110)	(n = 50)	
suPAR (˂2 ng/mL) *	7.25 (2.1−15)	7.55 (3.5−15)	0.109
CRP (˂5 mg/L) *	28.1 (1−359)	93.5 (10−364)	<0.001
PCT (˂0.5 µg/L) *	0.1 (0−49.23)	0.16 (0.02−36.2)	0.115
NLCR (1.4–4) *	4.9 (0.1−39)	6.55 (1−59.2)	0.615
IL-6 (˂7 µg/L) *	24.4 (1.5−2777)	46.3 (2.4−5000)	0.248
DD (˂0.23 mg/L) *	0.35 (0.05−11.7)	0.43 (0.11−7.92)	0.610
SARS-CoV-2 IgG (<33.8 BAU/mL) *	1470 (4.81−2080) ***	609 (4.81−2080) ****	0.035

* Data are expressed as the medians (range); ** *p* values denote results of multiple logistic regression; *** For SARS-CoV-2 IgG Mild COVID-19 data from 109 patients were used; **** For SARS-CoV-2 IgG Moderate/Severe COVID-19 data from 49 patients were used; COVID-19—coronavirus disease 2019; suPAR—soluble urokinase plasminogen activator receptor; CRP—C-reactive protein; PCT—procalcitonin; NLCR—neutrophil-to-lymphocyte count ratio; IL—interleukin; DD—D dimer, SARS-CoV-2 IgG—IgG antibodies to SARS-CoV-2.

## Data Availability

The original contributions presented in this study are in the article. Further inquiries can be directed to the corresponding author.

## References

[1] Elshazli R.M., Toraih E.A., Elgaml A., El-Mowafy M., El-Mesery M., Amin M.N., Hussein M.H., Killackey M.T., Fawzy M.S., Kandil E. (2020). Diagnostic and prognostic value of hematological and immunological markers in COVID-19 infection: A meta-analysis of 6320 patients. PLoS ONE.

[2] Metlay J.P., Waterer G.W. (2020). Treatment of community-acquired pneumonia during the coronavirus disease 2019 (COVID-19) Pandemic. Ann. Intern. Med..

[3] Shah S., Shah K., Patel S.B., Patel F.S., Osman M., Velagapudi P., Turagam M.K., Lakkireddy D., Garg J. (2020). Elevated D-dimer levels are associated with increased risk of mortality in coronavirus disease 2019: A systematic review and meta-analysis. Cardiol. Rev..

[4] Backes Y., van der Sluijs K.F., Mackie D.P., Tacke F., Koch A., Tenhunen J.J., Schultz M.J. (2012). Usefulness of suPAR as a biological marker in patients with systemic inflammation or infection: A systematic review. Intensive Care Med..

[5] Lipinski M., Rydzewska-Rosolowska A., Rydzewski A., Cicha M., Rydzewska G. (2017). Soluble urokinase-type plasminogen activator receptor (suPAR) in patients with acute pancreatitis (AP)—Progress in prediction of AP severity. Pancreatology.

[6] Rovina N., Akinosoglou K., Eugen-Olsen J., Hayek S., Reiser J., Giamarellos-Bourboulis E.J. (2020). Soluble urokinase plasminogen activator receptor (suPAR) as an early predictor of severe respiratory failure in patients with COVID-19 pneumonia. Crit. Care.

[7] Altintas I., Eugen-Olsen J., Seppälä S., Tingleff J., Stauning M.A., El Caidi N.O., Elmajdoubi S., Gamst-Jensen H., Lindstrøm M.B., Rasmussen L.J.H. (2021). suPAR cut-offs for risk stratification in patients with symptoms of COVID-19. Biomark. Insights.

[8] Napolitano F., Di Spigna G., Vargas M., Iacovazzo C., Pinchera B., Spalletti Cernia D., Ricciardone M., Covelli B., Servillo G., Gentile I. (2021). Soluble urokinase receptor as a promising marker for early prediction of outcome in COVID-19 hospitalized patients. J. Clin. Med..

[9] Kyriazopoulou E., Panagopoulos P., Metallidis S., Dalekos G.N., Poulakou G., Gatselis N., Karakike E., Saridaki M., Loli G., Stefos A. (2021). An open label trial of anakinra to prevent respiratory failure in COVID-19. eLife.

[10] D’Alonzo D., De Fenza M., Pavone V. (2020). COVID-19 and pneumonia: A role for the uPA/uPAR system. Drug Discov. Today.

[11] Nusshag C., Rupp C., Schmitt F., Krautkraemer E., Speer C., Kaelble F., Tamulyte S., Bruckner T., Zeier M., Reiser J. (2019). Cell cycle biomarkers and soluble urokinase-type plasminogen activator receptor for the prediction of sepsis-induced acute kidney injury requiring renal replacement therapy: A prospective, exploratory study. Crit. Care Med..

[12] Sudhini Y.R., Wei C., Reiser J. (2022). suPAR: An inflammatory mediator for kidneys. Kidney Dis..

[13] Loonen A.J.M., Kesarsing C., Kusters R., Hilbink M., Wever P.C., van den Brule A.J.C. (2017). High pneumococcal DNA load, procalcitonin and suPAR levels correlate to severe disease development in patients with pneumococcal pneumonia. Eur. J. Clin. Microbiol. Infect. Dis..

[14] Geboers D.G., de Beer F.M., Boer A.M.T.D., van der Poll T., Horn J., Cremer O.L., Bonten M.J., Ong D.S., Schultz M.J., Bos L.D. (2015). Plasma suPAR as a prognostic biological marker for ICU mortality in ARDS patients. Intensive Care Med..

[15] Vassiliou A.G., Zacharis A., Vrettou C.S., Keskinidou C., Jahaj E., Mastora Z., Orfanos S.E., Dimopoulou I., Kotanidou A. (2022). Comparison of the mortality prediction value of soluble urokinase plasminogen activator receptor (suPAR) in COVID-19 and sepsis. Diagnostics.

[16] Huang M., Li L., Shen J., Wang Y., Wang R., Yuan C., Huang M., Jiang L. (2020). Plasma levels of the active form of suPAR are associated with COVID-19 severity. Crit. Care.

[17] Wlazel R.N., Szwabe K., Guligowska A., Kostka T. (2020). Soluble urokinase plasminogen activator receptor level in individuals of advanced age. Sci. Rep..

[18] Langkilde A., Jakobsen T.L., Bandholm T.Q., Eugen-Olsen J., Blauenfeldt T., Petersen J., Andersen O. (2017). Inflammation and post-operative recovery in patients undergoing total knee arthroplasty-secondary analysis of a randomized controlled trial. Osteoarthr. Cartil..

[19] Qin C., Zhou L., Hu Z., Zhang S., Yang S., Tao Y., Xie C., Ma K., Shang K., Wang W. (2020). Dysregulation of immune response in patients with coronavirus 2019 (COVID-19) in Wuhan, China. Clin. Infect. Dis..

[20] Villard O., Morquin D., Molinari N., Raingeard I., Nagot N., Cristol J.P., Jung B., Roubille C., Foulongne V., Fesler P. (2020). The plasmatic aldosterone and C-reactive protein levels, and the severity of COVID-19: The Dyhor-19 study. J. Clin. Med..

[21] Luan Y.Y., Yin C.H., Yao Y.M. (2021). Update advances on C-reactive protein in COVID-19 and other viral infections. Front. Immunol..

[22] Bivona G., Agnello L., Ciaccio M. (2021). Biomarkers for prognosis and treatment response in COVID-19 patients. Ann. Lab. Med..

[23] Chalupa P., Beran O., Herwald H., Kaspříková N., Holub M. (2011). Evaluation of potential biomarkers for the discrimination of bacterial and viral infections. Infection.

[24] Gonzalez L., Holman T., Wait D., Abenojar P. (2022). Experience with procalcitonin use during the COVID-19 pandemic. Eur. J. Intern. Med..

[25] Battaglini D., Lopes-Pacheco M., Castro-Faria-Neto H.C., Pelosi P., Rocco P.R. (2022). Laboratory biomarkers for diagnosis and prognosis in COVID-19. Front. Immunol..

[26] Tabassum T., Rahman A., Araf Y., Ullah M.A., Hosen M.J. (2020). Prospective selected biomarkers in COVID-19 diagnosis and treatment. Biomark. Med..

[27] Callaway E. (2022). What Omicron’s BA.4 and BA.5 variants mean for the pandemic. Nature.

